# LPAIV H9N2 Drives the Differential Expression of Goose Interferons and Proinflammatory Cytokines in Both *In Vitro* and *In Vivo* Studies

**DOI:** 10.3389/fmicb.2016.00166

**Published:** 2016-02-17

**Authors:** Hao Zhou, Shun Chen, Bing Yan, Hongjun Chen, Mingshu Wang, Renyong Jia, Dekang Zhu, Mafeng Liu, Fei Liu, Qiao Yang, Ying Wu, Kunfeng Sun, Xiaoyue Chen, Bo Jing, Anchun Cheng

**Affiliations:** ^1^Institute of Preventive Veterinary Medicine, College of Veterinary Medicine, Sichuan Agricultural UniversityChengdu, China; ^2^Avian Disease Research Center, College of Veterinary Medicine, Sichuan Agricultural UniversityChengdu, China; ^3^Key Laboratory of Animal Disease and Human Health of Sichuan Province, Sichuan Agricultural UniversityChengdu, China; ^4^Shanghai Veterinary Research Institute, Chinese Academy of Agricultural SciencesShanghai, China

**Keywords:** AIV H9N2, geese, antiviral response, cellular immune, viral distribution

## Abstract

Geese, as aquatic birds, are an important natural reservoir of avian influenza virus (AIV). To characterize the innate antiviral immune response against AIV H9N2 strain infection in geese as well as the probable relationship between the expression of immune-related genes and the distribution of viral antigens, we investigated the levels of immune-related gene transcription both in AIV H9N2 strain-infected geese and *in vitro*. The patterns of viral location and the tissue distribution of CD4- and CD8α-positive cells were concurrently detected by immunohistochemical staining, which revealed respiratory and digestive organs as the primary sites of antigen-positive signals. Average AIV H9N2 viral loads were detected in the feces, Harderian gland (HG), and trachea, where higher copy numbers were detected compared with the rectum. Our results suggested the strong induction of proinflammatory cytokine expression compared with interferons (IFNs). Notably, in most tissues from the AIV H9N2 strain-infected birds, IFNα and IFNγ gene transcripts were differentially expressed. However, inverse changes in IFNα and IFNγ expression after AIV H9N2 strain infection were observed *in vitro*. Taken together, the results suggest that AIV H9N2 is widely distributed in multiple tissues, efficiently induces inflammatory cytokines in the HG and spleen of goslings and inversely influences type I and II IFN expression both *in vivo* and *in vitro*. The findings of this study further our understanding of host defense mechanisms and the pathogenesis of the H9N2 influenza virus in geese.

## Introduction

Avian influenza virus, a member of the family Orthomyxoviridae, which includes 18 HA and 11 NA subtypes (Tong et al., 2013), has a wide host range, including different types of birds and mammals. Influenza viruses have negative-sense, single-stranded, segmented genomes that are encapsulated by envelopes containing surface proteins and RNA. AIV infection causes significant economic losses in the poultry industry, and specific highly virulent infectious strains may even pose a threat to public health. In mainland China, AIV H9N2 was first isolated from chickens in Guangdong Province in 1994 (Chen et al., 1994). In 1999, AIV H9N2 was isolated from humans for the first time in Hong Kong (Guo et al., 1999). AIV H9N2 acts as a donor of potential segments and has reassorted with multiple other subtypes to form novel influenza virus genotypes (Sun and Liu, 2015). As previously described, poultry hosts infected with AIV H9N2 can act as a fundamental incubator for novel emerging pandemic AIVs, resulting in only mild disease(Liu et al., 2014), and chicken AIV H9N2 has been reported to facilitate the genesis of novel H7N9 influenza viruses (Pu et al., 2015). Moreover, the H9N2 virus is reported to have evolutionary human-like receptor identity (Matrosovich et al., 2001) and has adapted to bind mammalian host receptors, which has changed its transmissibility from birds to humans (Lin et al., 2000; Saito et al., 2001; Butt et al., 2005). Most LPAIV strains residing in wild birds such as waterfowl cause mild or no symptoms. Although H9N2-subtype AIVs are considered to be low pathogenic epidemic viruses, their coinfection with other viral pathogens appears to be a potential threat in other birds and mammals. Moreover, the H9N2 virus has been reported to have the ability to cross species barriers and can infect different hosts, ranging from a variety of birds to mammalian species, suggesting a considerable threat to public health. Remarkably, there are a host of reports about the immune regulation induced by LPAIV in chickens (Xing et al., 2008; Cornelissen et al., 2012; Wang et al., 2015), ducks (Cornelissen et al., 2012), and turkeys (Umar et al., 2015). Chronologically, the expression levels of both interferon (IFN)α and IFNγ are similarly unregulated in chickens and ducks after AIV H5N1 infection (Liang et al., 2011). Chickens and ducks differ in activation of pattern recognition receptors (PRRs) and IFN responses after LPAIV infection *in vivo* and after antagonist stimulation *in vitro* (Cornelissen et al., 2012). However, no data are available on the innate antiviral immune response of geese against AIV H9N2 infection.

Aquatic birds, and especially ducks and geese, are considered to be a reservoir for AIV, whereas high AIV mortality and pathogenicity have been observed in chickens (Slemons et al., 1990; Swayne, 1997; Yoon et al., 2014). The cohabitation of geese with domestic poultry, including chickens and ducks, may be one of the underlying factors responsible for AIV transmission and incubation. It is therefore important to explore the immune response of geese against AIV H9N2, which may further our understanding of the role that geese play in the evolution and transmission of AIV. To date, little information is available on the cytokine-level innate host responses of geese infected by LPAIV H9N2. The molecular mechanisms of the goose immune system that eliminate AIV and goose survival after infection have long been appreciated but remain poorly understood. At present, chicken and duck immune responses against several avian viruses have been widely studied. It is imperative to understand goose immune responses to LPAIV H9N2 infection, which may contribute to the as-yet-undefined relationship between AIV H9N2 and geese. These gaps prompted us to characterize the clinical symptoms of and the immune response against AIV H9N2 infection in geese, including the viral distribution in tissues during infection, the detection of several immune-related cytokines [IFNα, IFNγ, interleukin (IL)1, IL6, and myeloid differentiation primary response gene 88 (MyD88)], and the distribution of CD4- and CD8α-positive cells. The results of this study are conducive to increasing our knowledge of the antigenicity, distribution, and histopathology of the AIV circulating in geese.

## Materials and Methods

### Avian Influenza Virus

The AIV strain used in this study was A/chicken/JS/C1/2008 (AIV H9N2 strain), which was kindly provided by the Shanghai Veterinary Research Institute, Chinese Academy of Agricultural Sciences. The virus stocks were propagated in specific pathogen-free (SPF) chicken eggs, and the copy number for AIV was measured to be 7.14 × 10^12.64^ copies/ml. The viral TCID^50^ was 10^–7.375^/0.2 ml.

### Experimental Design

The study was conducted with 10 three-day-old AIV-free (confirmed by PCR detection) Sichuan White goslings. Five goslings each were infected AIV H9N2 by oral administration (250 μl) or intranasal injection (250 μl) (TCID_50_: 10^–7.375^/0.2 ml). Five PBS-treated goslings served as controls. After infection, the challenged goslings were monitored daily for clinical signs of disease, and their body weights were measured. The two groups of goslings were maintained in different isolation units in a biosecure animal building and fed a commercial diet *ad libitum*. Both the PBS-treated and the virus-infected goslings (initially 3 days old) were sacrificed at 5 days post-infection (5 dpi). Different tissues, including the T, SP, BF, CT, HG, SI, P, R, LU, TR, and LI, were collected. The animal studies were approved by the Institutional Animal Care and Use Committee of Sichuan Agricultural University and followed the National Institutes of Health guidelines for the performance of animal experiments.

### Immunohistochemical and Histopathological Analyses

Samples of selected tissues were immersion fixed in 4% paraformaldehyde, dehydrated, embedded in paraffin and sectioned. Immunohistochemical staining was slightly adjusted and conducted according as previously described (Chen et al., 2015). Mouse anti-duck monoclonal CD4 antibody (AbD Serotec MCA2478) and mouse anti-goose polyclonal CD8α antibody (provided by our laboratory) were diluted to 1:100. Rabbit polyclonal anti-influenza A H9N2 HA antibody was purchased from Sino Biological Inc. (Shanghai, China) and diluted to 1:200. Sections were subsequently incubated with biotinylated goat anti-mouse or goat anti-rabbit immunoglobulin G (IgG) (Biotin-Streptavidin HRP Detection Systems, ZSGB-BIO, Beijing, China) as the secondary antibody for 15 min at 37°C. The intensity of the immunoreactivity was subjectively scored using the following system according to prior work (Chen et al., 2009): no detectable antigen (–); weak, antigen was faintly detected (+); moderate, antigen was readily detected (++); and strong, antigen staining was intense (+++). Tissue samples were concurrently stained with haematoxylin and eosin (HE) prior to capture under a light microscope to assess the histopathology.

### Cell Culture

Peripheral blood mononuclear cells were isolated and purified from healthy adult geese. The PBMCs were grown overnight in RPMI supplemented with 10% fetal bovine serum, 100 U/ml penicillin, and 100 μg/ml streptomycin at 37°C and 5% CO_2_ at a cell density of approximately 1.0 × 10^7^ cells/well. The agonists and the indicated AIV H9N2 virus (50 μl) (TCID_50_: 10^–7.375^/0.2 ml) were then added to each well. The final concentration of poly IC (30 μg/ml) agonist (positive control) was added into medium. The cells were harvested for total RNA isolation at 6 h after stimulation, with PBS-treated wells serving as controls.

### RNA Isolation and Complementary DNA Preparation

Total RNA was isolated from selected tissues using RNAiso Plus reagent (Takara Bio, Otsu, Japan). The quantity and integrity of the RNA in each sample were determined using a NanoDrop 2000 (Thermo, Waltham, MA, USA). Complementary DNA (cDNA) was synthesized using a 5X All-In-One RT MasterMix Reagent Kit in accordance with the manufacturer’s instructions (Applied Biological Materials, Richmond, BC, Canada). Finally, the samples were stored at –80°C until use.

### Real-Time Quantitative PCR

The expression of IFN and inflammatory cytokine mRNA in leukocytes isolated from goose peripheral blood and tissues was detected by relative real-time quantitative PCR (qRT-PCR). The expression of goose immune-related genes was analyzed and selected based on specificity, as determined according to dissociation curves. The primer sequences are listed in **Table 1**. Real-time PCR was performed using the Bio-Rad CFX96 Real-Time Detection System (Bio-Rad, USA). Target gene expression was normalized to β-actin, a constitutively expressed endogenous control gene. Real-time PCR conditions and protocols were carried out as previously described (Zhou et al., 2015).

**Table 1:** List of primers used in this study and their sequences.

Primer name	Nucleotide sequence
IFNα (F)	CAGCACCACATCCACCAC
IFNα (R)	TACTTGTTGATGCCGAGGT
IFNγ (F)	TGAGCCAGATTGTTTCCC
IFNγ (R)	CAGGTCCACGAGGTCTTT
IL1β (F)	TCCGCCAGCCGCAAAGTG
IL1β (R)	CGCTCATCACGCAGGACA
IL6 (F)	AAGTTGAGTCGCTGTGCT
IL6 (R)	GCTTTGTGAGGAGGGATT
MyD88 (F)	CGTCTTTGATCGGGATGTCT
MyD88 (R)	AATCACATTCGTCGCTTTCC
β-actin (F)	CCGTGACATCAAGGAGAA
β-actin (R)	GAAGGATGGCTGGAAGAG
M (F)	TTCTAACCGAGGTCGAAAC
M (R)	AAGCGTCTACGCTGCAGTCC
Uni12M	AGCRAAAGCAGG (R = A/G)

### Detection of Viral Replication in the Feces and Tissues of Infected Goslings

To examine the shedding titre in cloacal swabs in the later part of the digestive system, the M gene of AIV H9N2 was amplified and then subcloned into the pMD18T vector (Takara, Japan) to prepare the reference DNA templates for qRT-PCR. The recombinant plasmid was serially diluted from 10^–1^ to 10^–8^ to establish the standard curve. The primer sequences for the M gene used in the present analysis are given in **Table 1**. The program parameters consisted of initial denaturation at 95°C for 10 min, followed by 40 cycles at 95°C for 15 s and 60°C for 1 min. A melting curve analysis was performed at the end of the reaction to assess the specificity of the PCR amplification. cDNA was synthesized from the RNA sample using the Uni12M primer. The cDNA from the cloacal feces and the viral cDNA from tissues were used as templates for detection, and each sample was analyzed in triplicate.

### Statistical Analysis

Data are indicated as the mean and standard deviation. The statistical analysis was performed on single and paired samples, as appropriate, by applying Student’s *t*-test. A P value less than 0.05 was considered to be statistically significant.

## Results

### Gross and Histopathological Examination

**FIGURE 1:**
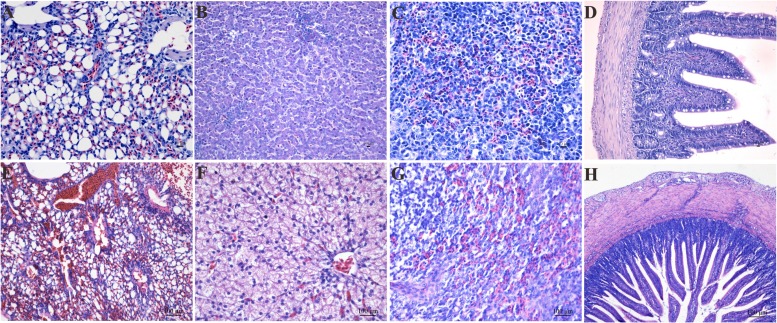
**The histological changes in the LU **(E)**, LI **(F)**, SP **(G)**, and SI **(H)** in the AIV H9N2 strain-infected goslings at 5 dpi.** Control groups were shown in **(A–D)**. The histological sections were stained with HE. Hyperaemia and congestion in the LU as well as cellular swelling, congestion, hepatic sinusoid disappearance, and suffuse hemorrhage in the SP were observed. Moderate pathological alterations in the SI were also observed.

In this study, we carried out infection with an LPAIV strain [A/chicken/JS/C1/2008 (AIV H9N2)]. The AIV H9N2-infected goslings did not show any obvious difference in weight gain or daily feeding at 5 dpi compared with the control group. The AIV H9N2-infected goslings showed slight weakness, a loss of appetite, and loose stool excretion. Several of the goslings like to squat together, whereas other goslings were unwilling to stand and remained close to their counterparts. A histopathological analysis showed obvious pulmonary hyperaemia and congestion (**Figures 1A,E**); cellular swelling, congestion, and hepatic sinusoid disappearance (**Figures 1B,F**); suffuse hemorrhage in the SP (**Figures 1C,G**); and moderate pathological manifestation in the SI (**Figures 1D,H**).

### Immunohistochemical Staining

**FIGURE 2:**
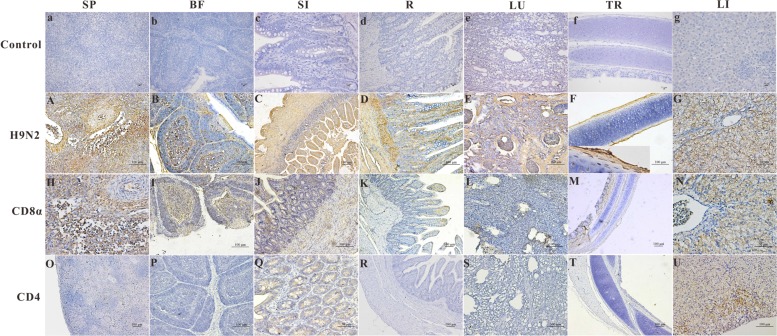
**The location of the AIV H9N2 antigen and goose CD4 and CD8α molecules in several tissues at 5 dpi.** The protein locations in the different tissues from AIV H9N2-infected birds were detected by IHC assay (Negative control group: **a–g**). Viral positive signals and cells positive for CD4 or CD8α antigen appeared dark brown using immunohistochemical staining. SP: SP **(A,H,O)**, BF: BF **(B,I,P)**, SI: SI **(C,J,Q)**, R: R **(D,K,R)**, LU: lung **(E,L,S)**, TR: TR **(F,M,T)**, LI: LI **(G,N,U)**.

The distribution and intensity of the immunoreactivity for the AIV antigen in paraformaldehyde-fixed, paraffin-embedded tissue sections after infection, as determined by IHC, are shown in **Figure 2** (Control group, **Figures 2A–G**). The AIV antigen was mostly strongly distributed in the SP (+++) (**Figure 2A**), SI (+++) (**Figure 2C**), R (+++) (**Figure 2D**) and lung (+++) (**Figure 2E**); was moderately detected in the BF (++) (**Figure 2B**) and the LI (++) (**Figure 2G**); and was faintly detected in the TR (+) (**Figure 2F**). Importantly, the antigen was readily and widely distributed in the mucous membrane epithelium and myofibrocytes of the intestines (+++) (**Figure 2C**) and in the follicle-associated epithelium, corticomedullary arch-forming cells, and epithelial reticular cells of the BF (++) (**Figure 2B**) as well as in the pseudostratified columnar epithelium of the TR (+) (**Figure 2F**). In goslings 5 dpi, CD8α-positive cells were found scattered throughout the SP (+++) (**Figure 2H**) and were most prevalent in the medulla and epithelial reticular cells of the BF (++) (**Figure 2I**), the hepatocytes in the LI (++) (**Figure 2N**), the pseudostratified columnar epithelium of the TR (+) (**Figure 2M**) as well as in the SI (+) (**Figure 2J**). These cells were also faintly detected in the LU (**Figure 2L**) and in the villi of the R (+) (**Figure 2K**). Moreover, CD4-positive cells could be detected in the mucous membrane epithelium of the SI (+) (**Figure 2Q**) and in the hepatocytes and mesenchymal cells of the LI (++) (**Figure 2U**) and were faintly detected in the SP (**Figure 2O**), LU (**Figure 2S**), R (**Figure 2R**), TR (**Figure 2T**) and BF (+) (**Figure 2P**).

### The Effects of AIV H9N2 on IFN and Proinflammatory Cytokine Expression *In Vivo*

**FIGURE 3:**
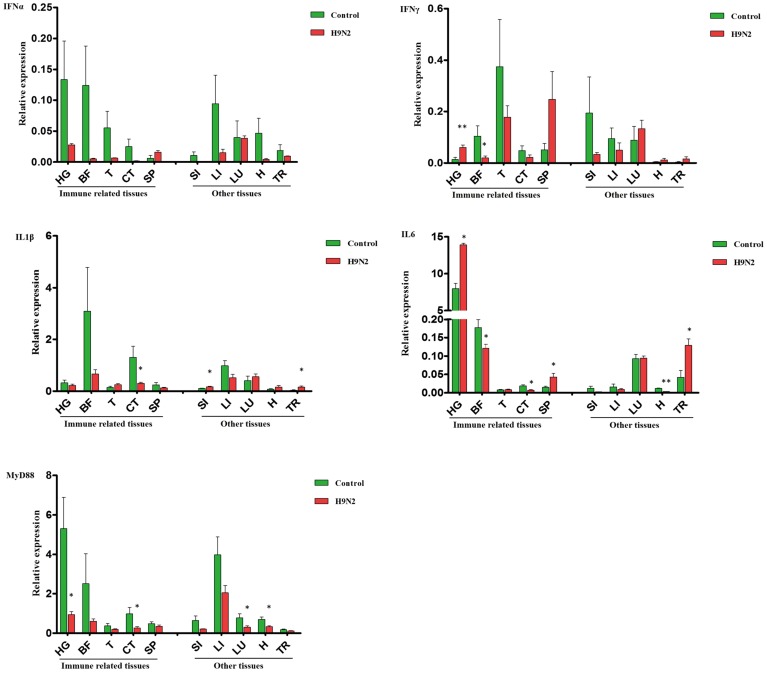
**A comparative analysis of immune-related genes, including IFNα, IFNγ, IL1β, IL6, and MyD88, in gosling tissues at 5 dpi.** Gene transcription levels in goose tissues were detected by qRT-PCR, and goose β-actin was amplified as an internal control. HG: Harderian gland, BF: BFbursa of Fabricius, T: thymus, CT: caecal tonsils, SP: spleen, SI: small intestine, LI: liver, LU: lung, H: heart, TR: trachea. mRNA expression was normalized using an internal control. The data are expressed as the mean ± SEM (*n* = 4), and the difference between the agonist-treated cells and the mock-treated cells was analyzed with a *t*-test. Groups denoted by one star (^∗^) represent a significant difference at *P* < 0.05, and groups denoted by two stars (^∗∗^) represent a significant difference at *P* < 0.01.

Inconsistent changes in immune effector expression were detected in various goose tissues. In the selected immune-related tissues (except the SP), IFNα gene expression was relatively lower in the AIV H9N2-infected group, whereas IFNγ transcript expression was increased in the HG (*P* < 0.01) and SP. A negative trend in other immune-related tissues was found, especially in the BF (*P* < 0.05) (**Figure 3**). Moreover, the IFNα was repressed in the BF at 7 dpi, but not at 1 and 3 dpi (**Supplementary Figure S1**). In other tissues, there was no significant difference in IFNα and IFNγ expression, but a negative trend for these IFNs was generally observed in the LI (**Figure 3**). Goose IL1β and IL6 gene expression was dramatically reduced in CT (P < 0.01); these interleukins were moderately expressed in the LI and LU and minimally expressed in the SI and T of infected goslings. As observed in **Figure 3**, IL6 was strongly upregulated in the HG (*P* < 0.01), SP (*P* < 0.01), and TR (*P* < 0.01) compared with the controls. The expression of goose MyD88 was inhibited to varying degrees in both primary immune tissues and other tissues.

### The Effects of AIV H9N2 on IFN and Proinflammatory Cytokine Expression *In Vitro*

**FIGURE 4:**
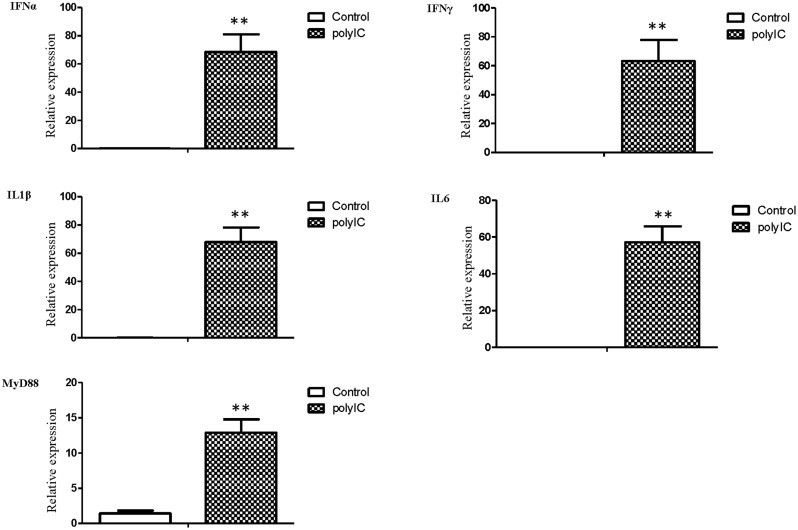
**The relative transcription levels of immune-related genes in poly IC (30 g/ml) treated goose PBMCs at 6 h post stimulation.** IFNα, IFNγ, IL1β, IL6, and MyD88 mRNA expression was normalized using an internal control. The data were expressed as the mean ± SEM (*n* = 4). The groups denoted by two stars (^∗∗^) represent a significant difference at *P* < 0.01.

**FIGURE 5:**
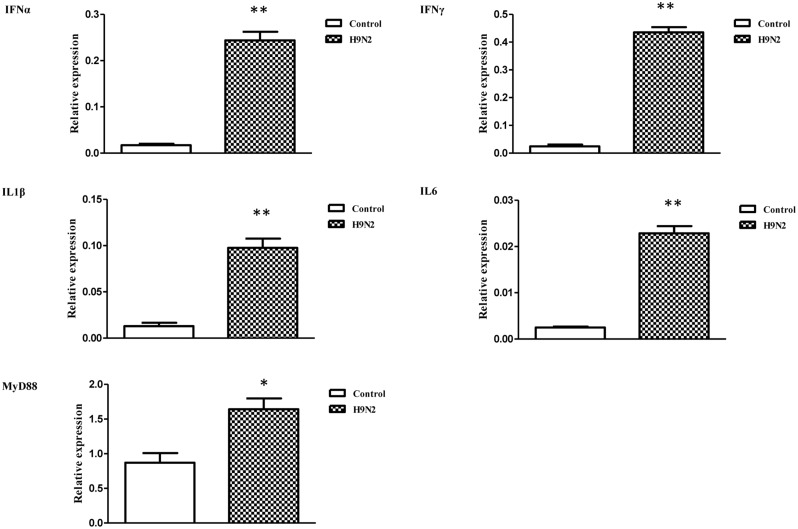
**The relative transcription levels of immune-related genes in AIV H9N2-treated goose PBMCs at 6 h post-infection.** IFNα, IFNγ, IL1β, IL6, and MyD88 mRNA expression was normalized using an internal control. The data were expressed as the mean ± SEM (n = 4), and the difference between the agonist-treated cells and the mock-treated cells was analyzed with a *t*-test. Groups denoted by one star (^∗^) represent a significant difference at *P* < 0.05, and groups denoted by two stars (^∗∗^) represent a significant difference at *P* < 0.01.

The mRNA transcript levels of goose interferons and pro-inflammatory cytokines were measured to quantify the response of goose PBMCs to poly IC (30 μg/mL). After 6 h incubation, poly IC induced a significant up-regulation of goose IFN-α (*P* < 0.01) and IFN-γ (*P* < 0.01), IL-1β (*P* < 0.01), IL-6 (*P* < 0.01), as well as MyD88 (*P* < 0.01) (**Figure 4**). In the AIV H9N2-infected goose PBMCs (**Figure 5**), elevated levels of IFNα and IFNγ were found after 6 h of stimulation (*P* < 0.01 and *P* < 0.01, respectively). Proinflammatory cytokine transcripts, including IL1β and IL6, were significantly upregulated at 5 dpi (*P* < 0.01 and *P* < 0.01, respectively). Consistent with this, the MyD88 transcript was also significantly upregulated in the infected cells (*P* < 0.05).

### Viral Copies in the Feces and Tissues of Infected Goslings

**FIGURE 6:**
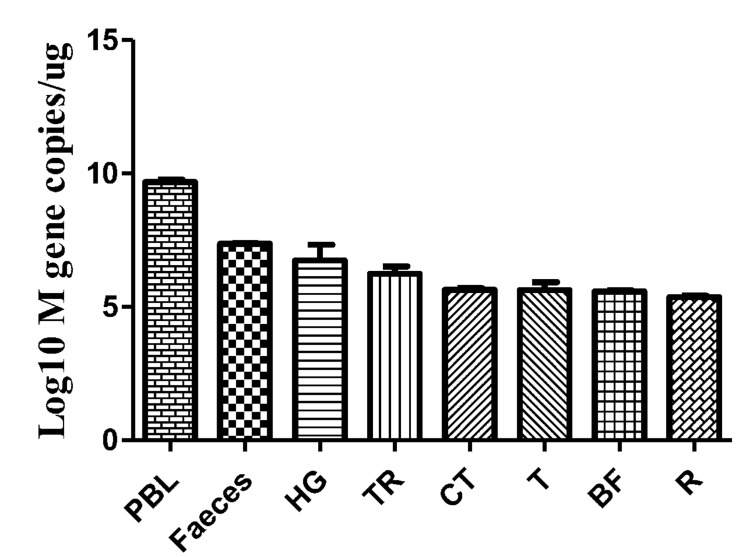
**Viral copies in the selected tissues from AIV H9N2-infected geese at 5 dpi.** The efficiency of AIV M gene amplification by qRT-PCR was 100.1%, and *R*^2^ was 0.998. The average viral copy number showed an evident difference between individual samples. The average number of viral copies was detected in the peripheral blood (10^9.68^ copies/μg), feces (10^7.36^ copies/μg), HG (10^6.74^ copies/μg), TR (10^6.24^ copies/μg), CT (10^5.64^ copies/μg), T (10^5.63^ copies/μg), BF (10^5.59^ copies/μg), and R (10^5.37^ copies/μg). The data are expressed as the mean ± SEM (*n* = 3).

Faeces were collected from the intestinal tracts of geese, and peripheral blood and tissues, including the HG, TR, CT, T, BF, and R, were collected. The viral copy number was then detected by qRT-PCR. The efficiency of M gene amplification was 100.1%, and *R*^2^ was 0.998. After viral nucleotide extraction, the viral copy number showed an evident difference between individual samples. Viral copies were highly detected in the peripheral blood (10^9.68^ copies/μg), feces (10^7.36^ copies/μg), HG (10^6.74^ copies/μg), TR (10^6.24^ copies/μg), and CT (10^5.64^ copies/μg) (**Figure 6**).

## Discussion

Large pools of AIV cover a variety of subtypes of wild water birds, including wild ducks and geese. The prevention and treatment of AIV remain a challenge because of our incomplete understanding of the restriction and dissemination of AIV in aquatic birds as well as the lack of comprehensive information regarding innate immune regulation in birds. Although the pathogenesis of AIV in geese has not been fully elucidated, there is evidence that water birds act as mixed reservoirs of currently circulating and pervasive avian influenza strains. Specifically, certain avian H9N2 viruses harbor receptor-binding characteristics similar to those of human strains of the virus, which are a contributing factor to increases in both the potential reassortment of AIV and potential transmission between diverse species (Lin et al., 2000).

The results of our study suggest that during the early infection phase, geese showed moderate clinical symptoms, with no appetite and moderate diarrhea. The mechanisms accounting for how LPAIV H9N2 strains infect avians remain to be explored. Notably, geese are natural reservoirs of AIV and can survive after infection by AIV strains without obvious clinical signs. The differences in immune-related gene expression between duck and chicken PBMCs infected by AIV H11N9 are characterized by asymptomatic and lasting infections in ducks and rapid clearance in chickens (Adams et al., 2009). Our study indicated that the moderate to severe lesions in the LI, LU, and SP of infected goslings, as shown by HE staining, may be due to the fact that low-virulence AIV strains can also lead to pathological manifestations in susceptible tissues but cause only obscure clinical signs. Initially infecting poultry, avian influenza A (H9N2) viruses have been sporadically identified in mammals, including pigs and humans (Yoon et al., 2014). Chicken LPAIVs mainly proliferate in the LUs and intestine (Spickler et al., 2008). Compared with mammalian model systems, little is understood about the distribution and location of the AIV antigen in geese infected with LPAIVs (H9N2). Due to limited information about the antigen state, how and why aquatic birds can survive AIV infections also remain unclear. Herein, the H9N2 strain was not confined to the intestinal tract alone, but rather was also observed in the upper respiratory organs of the geese, including the LUs and TR. Moreover, high viral copy numbers were detected in the TR and feces, which suggests that the virus was widely distributed in the respiratory and digestive systems. The main viral target cells are epithelia, such as intestinal epithelial cells, which may explain the disorders of the intestinal epithelium’s secretory and absorptive functions. The BF is critical for normal development of the B lymphocytes responsible for antibody production. CD4 and CD8 T lymphocytes are involved in the process of viral clearance. In this context, the goose SP is an important lymphoid organ in which CD4-positive cells are scattered. Importantly, AIV H9N2 was also largely found in the SP and BF in the current study, which may explain the disruption of cellular and humoral immunity in the host. Additionally, influenza virus infection has been reported to increase susceptibility to secondary infections (Metzger and Sun, 2013). Goose IFNα was impressed in BF at both 5 and 7 dpi. Normally, CD8α-positive signals are only slightly detected in the SP and BF, whereas CD4-positive signals are easily observed in the SP (Chen et al., 2015). However, after AIV H9N2 infection in the present study, CD8α-positive cells were readily detected in the SP, BF, SI, and TR, whereas CD4-positive signals were hardly detected in the SP of infected goslings; this suggests that CD8 T cells may be activated, whereas CD4 T cells may be suppressed to a certain extent. Alternatively, CD8 T cells may play a predominant role in AIV host defense. With these data in mind, we have attempted to elucidate the molecular mechanism(s) against AIV H9N2 infection in geese. Intriguingly, viral distribution was prevalent, and the number of viral copies was highest in the peripheral blood, demonstrating that the virus can circulate via the circulatory system. This finding is consistent with the results of previous studies that found that high cloacal viral loads as well as viral RNA in the blood during the early phase of H9N2 infection in immunocompromised chickens (Kwon et al., 2008). Furthermore, AIV can replicate at low levels in ducks for around a month and be shed intermittently (Higgins et al., 1987). The expression levels of IFNs are upregulated significantly in chickens and ducks after AIV H5N1 infection (Liang et al., 2011). The duck IFNγ mRNA was strongly up-regulated in the LU and bursa infected by LPAIV H7N1 (Cornelissen et al., 2012). However, the IFN response of H9N2 strain infected goslings was different from previous results. In the present study, the induction of IFN *in vivo* may have correlated with viral loads; therefore, AIV H9N2 may persist in gosling to evade the IFN response.

Toll-like receptors (TLRs) can function as sensors to safeguard the body from danger signals. Viral recognition relies on PRRs; for example, the ligands for TLR3 are dsRNAs derived from viruses, and the ligands for TLR7 are ssRNAs derived from RNA viruses. TLR recognition activates downstream signal transduction to activate nuclear factor (NF)-κB or IFN regulatory factor (IRF) 3/7, which ultimately induces IFN and inflammatory cytokine production (Takeuchi et al., 2004; Kawai and Akira, 2005). AIV may be recognized by the endosomal receptor TLR7 and the cytoplasmic sensor retinoic acid-inducible gene (RIG)-I. Previous reports have indicated that host responses against AIV differ depending on the viral strain (Sutejo et al., 2012). In the present report, increased secretion of proinflammatory cytokines (such as IL1β and IL6) was observed. The drastic increase in IL6 in geese may have been due to inflammatory pathological changes in the LU, where the virus replicated. Although the levels of proinflammatory cytokines were largely increased in most tissues of the infected goslings, type I and type II IFN responses were weak and were differentially regulated in distinct tissues. Previous data have demonstrated that suppression of IFN and IFN-inducible genes was observed in chicken macrophages (Xing et al., 2008). The stimulation of goose blood monocytes with poly IC agonists alone can produce stronger T helper cell 1-biased cytokine responses, including the upregulation of IFNα, IFNγ, and IL6. The immune response induced by AIV and activated by poly IC in goose PBMCs shows the same trend. Interestingly, we observed significant upregulation of proinflammatory cytokines (IL1β and IL6) and IFNs by the AIV H9N2 strain *in vitro*. The expression of IFNα observed in the *in vivo* study was not confirmed in the H9N2 virus-infected cells; that is, the H9N2 strain did not behave similarly with respect to IFN expression *in vivo* and *in vitro*. It appears that the host response against this strain may vary depending on the type of host cell. Additionally, these findings reflect the complex pathogenesis of AIV. There was also no significant change in IFN gene expression levels in certain tissues *in vivo* post-infection with the H9N2 virus. The reasons for this may be that the cytokines were transiently expressed and that the samples differed between individuals. In our studies, IFNα was suppressed to a certain extent *in vivo*, which was in accordance with the fact that AIV can inhibit IFN-mediated antiviral responses (Garcia-Sastre, 2001). One hypothesis regarding the minor suppression of IFNs is that the non-structural (NS) gene is involved in the transient resistance of AIV to IFN action due to the inactivation of protein kinases (Sekellick et al., 2000). The NS1 protein of influenza A virus can function as an IFN antagonist, preventing the host IFN antiviral response during viral infection. *In vivo*, NS1-knockout influenza A viruses effectively trigger IFN activity (Garcia-Sastre et al., 1998). Although type I and type II IFNs were differently repressed in most immune-related tissues *in vivo* in the current study, inflammatory cytokines (e.g., IL6) showed an upward trend in certain tissues. Thus, the host may become more susceptible to secondary infection by other pathogens due to the inhibition of IFN induction. This may be the underlying explanation for the substantial innate immune system changes observed in geese after AIV H9N2 infection and propagation. These findings suggest that certain specific subtypes of the AIV H9N2 virus may participate in the negative modulation of the host adaptive immune response in aquatic birds. Further details of the real-life complex mechanisms of host defense and the immune responses of AIV H9N2-infected geese should be elucidated.

In our study, LPAIV H9N2 infection in aquatic birds resulted in obscure superficial clinical syndromes but a wide antigen distribution, causing respiratory, and digestive dysfunction. Extensive virus replication was primarily observed in the goose respiratory system and intestinal tissues. In particular, the SP, SI, R, LUs, and peripheral blood all showed high viral copy numbers, indicating lasting and asymptomatic infection in the geese. Furthermore, goose IFNs were differentially suppressed by AIV H9N2 infection *in vivo*, which is different from the cytokine expression *in vitro*. The results of this study not only have addressed AIV H9N2 antigen distribution in geese but also have expanded our fundamental understanding of the goose immune response against AIV H9N2 infection.

## Author Contributions

ZH performed most of the experiments with the help of YB and CS; ZH, WM, JR, and ZD performed the data analysis; CS and CA designed the study; WM, JR, ZD, LM, LF, YQ, WY, SK, CS, and CA supervised the study and provide the materials. ZH and CS wrote the paper. CH, CX, and JB provided the viral strain and gave the experimental support.

## Conflict of Interest Statement

The authors declare that the research was conducted in the absence of any commercial or financial relationships that could be construed as a potential conflict of interest.

## Supplementary Material

FIGURE S1 **Relative expression analysis of type I interferon (IFNα) in BF and SI of gosling at 1, 3, 7 dpi. IFNα gene transcription levels in goose tissues were detected by qRT-PCR, and goose β-actin was amplified as an internal control.** The data were expressed as the mean ± SEM (*n* = 4), and the difference between the control group and the virus-infected group was analyzed with a *t*-test. Groups denoted by one star (^∗^) represent a significant difference at *P* < 0.05, and groups denoted by two stars (^∗∗^) represent a significant difference at *P* < 0.01.

## ABBREVIATIONS

AIV: Avian influenza virus
BF: bursa of Fabricius
CT: caecal tonsils
HA: haemagglutinin
HG: Harderian gland
IHC: immunohistochemistry
LPAIV: low pathogenic avian influenza virus
LU: lung
LI: liver
NA: neuraminidase
P: pancreas
R: rectum
SP: spleen
SI: small intestine
T: thymus
TR: trachea
PBMCs: peripheral blood mononuclear cells
